# Abnormal performance of peroral endoscopic myotomy (POEM): a case misdiagnosed as achalasia of cardia

**DOI:** 10.1186/s13019-024-02688-w

**Published:** 2024-04-15

**Authors:** Wen-Quan Yu, Hui-Jiang Gao, Li-Xue Zhai, Yu-Cheng Wei

**Affiliations:** 1 Department of Thoracic Surgery, The Affiliated Yantai Yuhuangding Hospital of Qingdao University, Yantai, China; 2 Department of Thoracic Surgery, Affiliated Hospital of Qingdao University, 16 Jiangsu Road, Shinan District, Qingdao, Shandong Province China; 3 Department of Ultrasonography, Beijing Friendship Hospital, Capital Medical University, Beijing, China

**Keywords:** Pseudoachalasia, Peroral endoscopic myotomy, Achalasia

## Abstract

**Background:**

Pseudoachalasia is a rare disease that behaves similarly to achalasia (AC), making it sometimes difficult to differentiate.

**Case presentation:**

We report a case of 49-year-old male with adenocarcinoma of the gastroesophageal junction misdiagnosed as achalasia. No obvious abnormalities were found in his initial examinations including upper digestive endoscopy, upper gastrointestinal imaging and chest computed tomography (CT). During the subsequent introduced-peroral endoscopic myotomy (POEM), it was found that the mucosal layer and the muscular layer had severe adhesion, which did not receive much attention, delayed the clear diagnosis and effect treatment, and ultimately led to a poor prognosis for the patient.

**Conclusions:**

This case suggests that when patients with AC found mucosal and muscular adhesions during POEM surgery, the possibility should be considered that the lesion may be caused by a malignant lesion.

**Supplementary Information:**

The online version contains supplementary material available at 10.1186/s13019-024-02688-w.

## Background

AC is a primary esophageal dyskinesia disease of unknown etiology, with an annual reported incidence of about 1 in 100,000 worldwide [1], characterized by poor relaxation of the lower esophageal sphincter and loss of peristalsis of the esophagus [2]. Pseudoachalasia, also known as secondary achalasia, is a dysphagia syndrome caused by secondary etiology with achalasia-like symptoms. Due to similar clinical manifestations and examination results, sometimes it is difficult to distinguish pseudoachalasia from AC, which causing misdiagnosis and mistreatment can reduce the quality of life and survival time of patients [3].Therefore, it is particularly important to enhance the knowledge of clinicians on the two diseases and improve the accuracy of clinical diagnosis.

## Case presentation

**Fig. 1:**
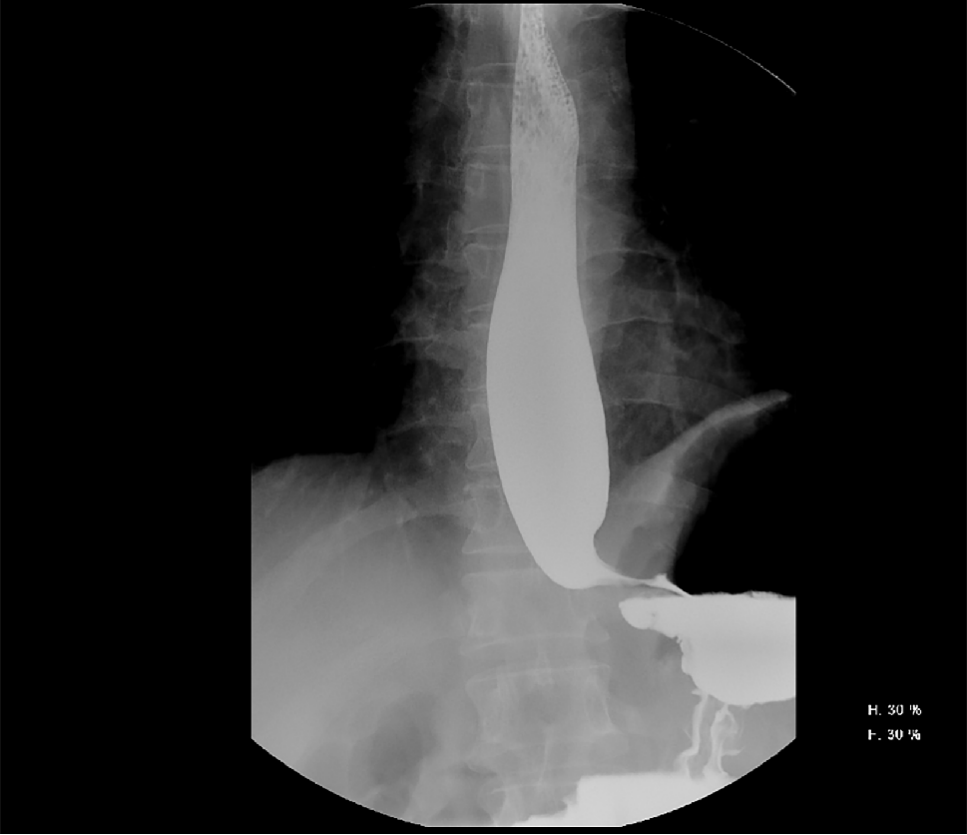
Upper Gastrointestinal Imaging. upper gastrointestinal imaging showed the smooth tapering distal esophagus (“Bird’s beak” appearance) and dilated esophagus with barium

**Fig. 2:**
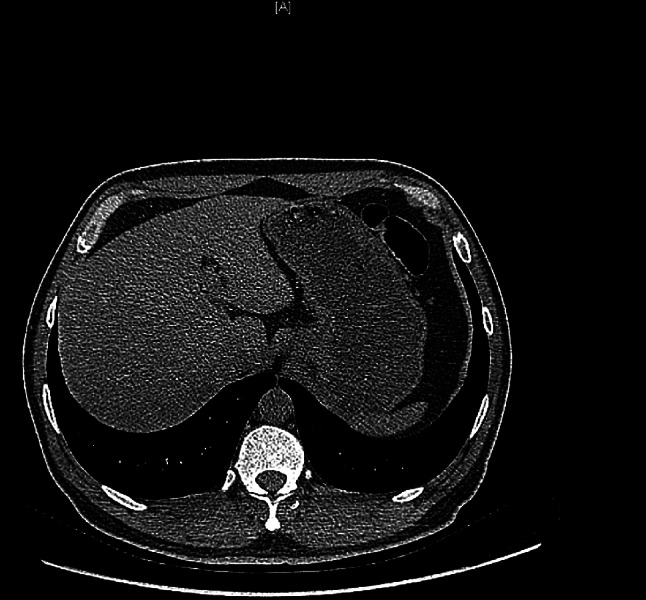
Computed Tomography of upper abdomen. CT showed dilated esophagus and insignificant thickening of the lateral wall of the cardia, beyond that there isn’t any other abnormality

**Fig. 3:**
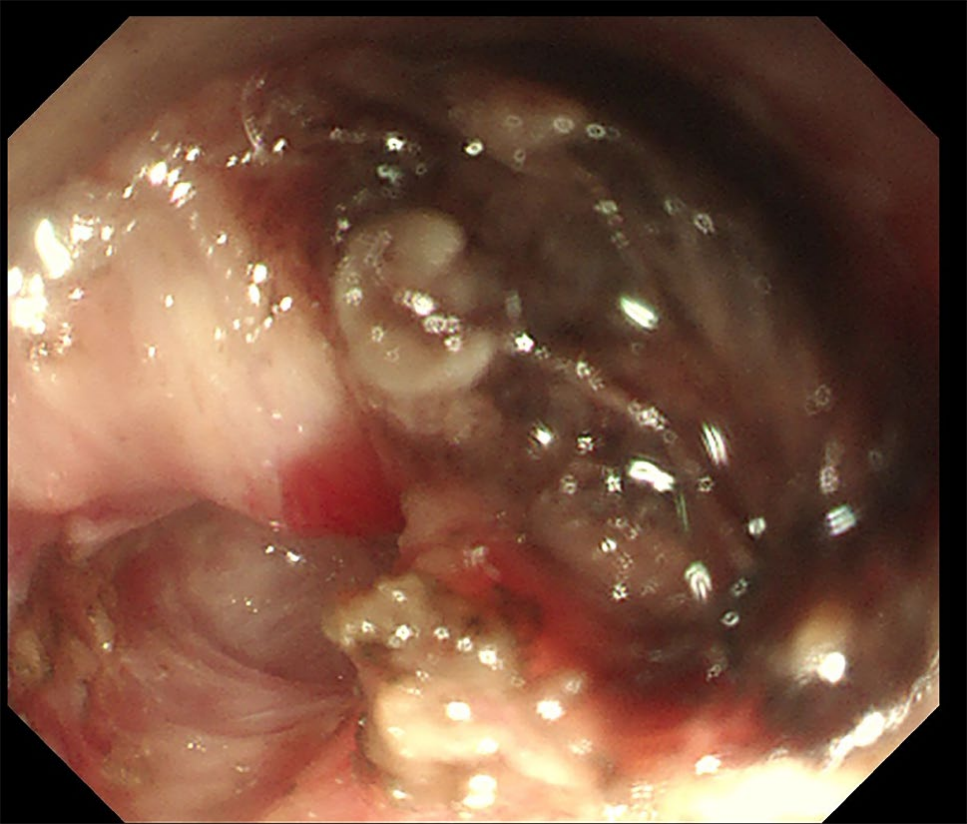
Endoscopic images in peroral endoscopic myotomy. The endoscopic image showed the condition of the esophagogastric junction during the peroral endoscopic myotomy: the mucosa and muscularis had serious adhesion, and an exploratory separation was performed resulting in slight bleeding

A 49-year-old male patient was referred to the local hospital for complaining of gradual aggravated dysphagia with no obvious inducement one month ago, without hematemesis, hematochezia, and no significant weight loss. The patient underwent two upper digestive endoscopy, one showed smooth esophageal mucosa and good cardia expansion, the other showed fluid retention at the gastroesophageal junction, unclear mucosa, and narrowing of the cardia, but the endoscope could reluctantly pass into the gastric cavity. Two upper gastrointestinal imaging showed the smooth tapering distal esophagus (“Bird’s beak” appearance) and dilated esophagus with barium. (Fig. 1) CT showed dilated esophagus and insignificant thickening of the lateral wall of the cardia, beyond that there isn’t any other abnormality. (Fig. 2) A diagnosis of achalasia was obtained combining the above clinical evidence. Then the doctor communicated with the patient and his family to obtain their consent and scheduled for POEM. After general anesthesia, upper gastrointestinal endoscopy was used to explore the whole process of esophagus: stenosis appeared at 40 cm from the incisor, and the surface mucosa was smooth. After the mixed solution of adrenaline, normal saline and methylene blue was injected into the submucosa 30 cm from the incisor, a longitudinal 1.2 cm mucosal incision was made to establish a tunnel for submucosal stripping under endoscopic-assisted. When the endoscope reached 39 cm from the incisor in the tunnel under the mucosa, it was found that the mucosa and muscularis had serious adhesion, and the two layers could not be separated. (Fig. 3) After the incision was closed with a titanium clip the operation was terminated. Postoperatively, the patient occurred esophageal fistula and mediastinal abscess, which was treated by mediastinotomy and drainage. At this time, the doctor checked the blood tumor markers: CEA, CA-125, CA-242, and AFP were all normal. Cardioplasty was performed after two months of infection completely control. At the same time, the scars near the cardia were removed for pathological examination, showing that it was highly differentiated adenocarcinoma. Two months later, he was referred to our hospital and planned to undergo radical surgery for gastroesophageal junction adenocarcinoma. Preoperative, we performed a complete oncology-related examination and there was no evidence of metastases other than the primary tumor and performed the operation with the consent of the patient. During the operation, we found that the cardia and the surrounding tissues including lung tissue, diaphragm, omentum, and left lobe of the liver were very tightly adhered due to multiple operations. The operation was difficult, but eventually we completed the operation. Postoperative pathology: (cardia) moderately differentiated adenocarcinoma. (p-T4aN1M0G2). The patient recovered smoothly and was transferred to the oncology department to continue treatment, but he died ultimately after 4 months due to tumor recurrence.

## Discussion and conclusions

AC is a primary esophageal motor disorder, and the esophageal motility testing (high-resolution manometry) is the gold standard for the diagnosis of AC, but some studies have pointed out that its role in identifying pseudoachalasia is limited [4, 5]. There are many causes of pseudoachalasia including benign and malignant diseases, and the proportion of pseudocardiac achalasia caused by malignant tumors exceeds 50% [6, 7]. Most of them can be diagnosed through upper gastrointestinal angiography, multiple gastroscopy and biopsy, chest CT and other auxiliary examinations to find malignant signs to confirm the diagnosis; but for those patients with early lesions and negative auxiliary examinations, the diagnosis is often difficult. In this case, no malignant signs were found on the endoscope and upper gastrointestinal angiography before POEM, and the thickening of the cardia on the lesser curvature side reported by chest CT was not obvious, which can be explained by contracture of the cardia sphincter [8]. During the POEM operation, it was found that the mucosa was tightly adhered to the muscularis, which was not consistent with the intraoperative performance of conventional AC patients. A mucosal bleb was easily created by submucosal injection in general AC patients and the endoscope can be easily introduced into the submucosal area [9]. This is because AC, as a benign lesion, does not damage the anatomical structure of each layer of the esophagus wall, so that the mucosal layer and the muscular layer can be separated smoothly. Combined with the pathological results, we analyzed that the adhesion of the tissue was caused by the infiltration and growth of the malignant tumor under the mucosa and the destruction of the surrounding tissue structure, resulting in the scar of the surrounding tissue. At this time, if the diagnosis of pseudoachalasia is considered, the prognosis may be better after effective treatment after pathological results are obtained. Of course, the positive rate of pathological examination of specimens obtained under endoscopy is not 100%, but the signs in this POEM operation can give us a good reminder of possible malignant diseases. In addition, endoscopic ultrasound plays an important role in the differential diagnosis of two diseases. The American College of Gastroenterology recommends that esophageal ultrasound endoscopy (EUS) should be performed on patients with suspected pseudoachalasia, which can further find some invasive tumors. Some studies have pointed out that EUS can find smaller diameters submucosal cancer that any other examination can’t found out [2, 10]. But there is still some misdiagnosis rate with EUS. Sarra Oumrani’s study points out that the contribution of the EUS finding in patients with oesophageal dyskinesia was limited in their study, and did not alter patient management [11]. In a large series of 18 patients with pseudoachalasia, Ponds et al. reported that EUS alone helped making the diagnosis of pseudoachalasia in only two cases [5]. 

POEM is a new method for treating AC in recent years. Due to its good safety and effectiveness, it is more and more commonly used in the treatment of AC [12]. There have been report of patient with esophageal leiomyoma who have been definitely diagnosed and treat when performing POEM [13]. However, it’s rarely reported that cases of pseudoachalasia caused by malignant tumors accidentally discovered by POEM. Especially for cases which there is no obvious positive test result in the preoperative auxiliary examination, if serious adhesion between the mucosa and the muscularis is found during POEM, it is particularly important to remind clinicians a diagnosis idea: Adhesion might be caused by malignant diseases.

### Electronic supplementary material

Below is the link to the electronic supplementary material.

Supplementary Material 1

## Data Availability

The datasets during and/or analysed during the current study available from the corresponding author on reasonable request.

## Abbreviations

AC: Achalasia
POEM: Peroral endoscopic myotomy
EUS: Esophageal ultrasound endoscopy
