# Supplementary material for: Abnormal performance of peroral endoscopic myotomy (POEM): a case misdiagnosed as achalasia of cardia
Source: J Cardiothorac Surg. 2024 Apr 15;19:214. doi: 10.1186/s13019-024-02688-w (PMC11017660; doi:10.1186/s13019-024-02688-w)
Supplement: Supplementary file 1 — Supplementary Material 1 [file 13019_2024_2688_MOESM1_ESM.pdf]

CERTIFICATE OF ENGLISH EDITING

This is to certify that the manuscript entitled

Abnormal performance of Peroral endothelial myotomy (POEM): a case misdiagnosed as achalasia of the cardia

commissioned to us has been carefully edited by a native English-speaking editor of MogoEdit, and the grammar, spelling, and punctuation have been verified and corrected where needed. Based on this review, we believe that the language in this paper meets academic journal requirements. Please contact us with any questions.

Gang Zhang

Dr. Gang Zhang
Founder & CEO of MogoEdit

Date of Issue
December 6, 2022

Disclaimer: The changes in the document may be accepted or rejected by the authors in their sole discretion after our editing. However, MogoEdit is not responsible for revisions made to the document after our edit on **December 6, 2022**.

MogoEdit is a professional English editing company who provides English language editing, translation, and publication support services to individuals and corporate customers worldwide. As a company invested by the affiliate fund of Chinese Academy of Science, MogoEdit is one of the leading language editing service providers in China, whose clients come from more than 1000 universities and research institutes.
MogoEdit Website: http://en.mogoedit.com/
500+ native English editors: http://en.mogoedit.com/editors

make the world flatter

Mogo Internet Technology Co., LTD.
No. 57, 3rd Keji Road, Xi'an 710075, PR China +86 02988317483 support@mogoedit.com
